# Video‐assisted thoracoscopic surgery for ectopic mediastinal parathyroid adenoma

**DOI:** 10.1002/bjs5.50207

**Published:** 2019-08-19

**Authors:** K. E. Isaacs, S. Belete, B. J. Miller, A. N. Di Marco, S. Kirby, T. Barwick, N. S. Tolley, J. R. Anderson, F. F. Palazzo

**Affiliations:** ^1^ Department of Endocrine Surgery Imperial College Healthcare NHS Trust London UK; ^2^ Department of Radiology Imperial College Healthcare NHS Trust London UK; ^3^ Department of Anaesthesia Imperial College Healthcare NHS Trust London UK; ^4^ Department of Cardiothoracic Surgery, Hammersmith Hospital Imperial College Healthcare NHS Trust London UK; ^5^ Department of Surgery & Cancer Imperial College London UK

## Abstract

**Background:**

Primary hyperparathyroidism (PHPT), caused by an ectopic mediastinal parathyroid adenoma, is uncommon. In the past, when the adenoma was not accessible from the neck, median sternotomy was advocated for safe and successful parathyroidectomy. Video‐assisted thoracoscopic surgical (VATS) parathyroidectomy represents a modern alternative approach to this problem.

**Methods:**

Information on patients undergoing VATS was obtained from a specific database, including clinical presentation, biochemistry, preoperative imaging, surgical approach and patient outcomes. A comprehensive literature review was undertaken to draw comparisons with other publications.

**Results:**

Over a 2‐year period, nine patients underwent VATS parathyroidectomy for sporadic PHPT. Five patients had persistent PHPT following previous unsuccessful parathyroidectomy via cervicotomy, and four had had no previous parathyroid surgery. The median duration of surgery was 90 (range 60–160) min. Eight patients were cured biochemically, with no major complications. One patient required conversion to a median sternotomy for removal of a thymoma that had resulted in false‐positive preoperative imaging.

**Conclusion:**

With appropriate preoperative imaging, multidisciplinary input and expertise, VATS parathyroidectomy is an effective, safe and well tolerated approach to ectopic mediastinal parathyroid adenoma.

## Graphical abstract

The authors' unit recently adopted a thoracoscopic approach to mediastinal parathyroidectomy. Unit data for their case series of thoracoscopic parathyroidectomies are reviewed here, including preoperative workup and operative findings.

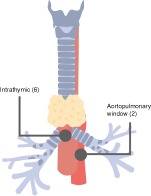

Recommended approach to mediastinal adenomas

## Abstract (es)

**Antecedentes:**

El hiperparatiroidismo primario (*primary hyperparathyroidism*, pHPT) causado por un adenoma paratiroideo ectópico mediastínico es infrecuente. Hace años, cuando un adenoma no era accesible por vía cervical se propugnaba una esternotomía media para efectuar una paratiroidectomía segura y con éxito. La paratiroidectomía por cirugía toracoscópica asistida por video (*video‐assisted thoracoscopic surgical*, VATS) es una alternativa moderna para el abordaje de esta patología.

**Métodos:**

La información de los pacientes tratados con VATS se obtuvo de una base de datos específica, incluyendo presentación clínica, bioquímica, radiología preoperatoria, abordaje quirúrgico y resultados de los pacientes. Se efectuó una revisión extensa de la literatura para efectuar comparaciones con otras publicaciones.

**Resultados:**

Durante un periodo de 2 años, 9 pacientes fueron tratados mediante paratiroidectomía por VATS debido a un pHPT esporádico, de los cuales 5 presentaban pHPT persistente después del fracaso de una paratiroidectomía por cervicotomía, mientras que los 4 restantes no habían sido operados previamente de cirugía paratiroidea. El tiempo medio operatorio fue de 101 minutos (rango 60‐160). Ocho pacientes se curaron bioquímicamente, sin ninguna complicación mayor. Un paciente precisó conversión a una esternotomía media para extirpar un timoma que había sido un falso positivo en la radiología preoperatoria.

**Conclusión:**

La paratiroidectomía por VATS es una intervención efectiva, segura y bien tolerada para la extirpación de un adenoma ectópico mediastínico, siempre y cuando se disponga de radiología preoperatoria adecuada, equipo multidisciplinar y experiencia.

## Introduction

Primary hyperparathyroidism (PHPT) is a dysregulation of bone and mineral metabolism caused by the inappropriately unsuppressed secretion of parathyroid hormone (PTH)^1^. It has an estimated prevalence 1–4 per 1000 of the general population, is more common in women, and its incidence increases with age^2^. Parathyroidectomy is the only definitive cure for PHPT, and curative surgery has been shown to increase bone mineral density and reduce renal calculi. It may also improve the neurocognitive symptoms described by many patients with PHPT^1^, ^3^, ^4^, ^5^, ^6^.

The superior parathyroid glands arise from the fourth branchial pouch, and the inferior parathyroid glands arise from the third branchial pouch with the thymus^7^, ^8^. During development, the glands descend inferiorly while maintaining an intimate association with their associated branchial pouch derivatives. The superior glands usually come to lie superior to the inferior thyroid artery and posterior to the recurrent laryngeal nerve, whereas the inferior parathyroid glands usually lie inferior to the inferior thyroid artery and anterior to the recurrent nerve^8^. Inferior parathyroid glands may lie anywhere along the thyrothymic tract, with up to 50 per cent found within the thymus or thyrothymic tongue^9^. Truly ectopic superior parathyroid glands are uncommon, but may be found in the middle or posterior mediastinum, or, rarely, in the aortopulmonary window^9^.

Ectopic mediastinal parathyroid adenomas are a recognized cause of persistent PHPT, and the cause of persistent hypercalcaemia in approximately 0.8 per cent of reoperative parathyroidectomies^10^. A large proportion of mediastinal parathyroid adenomas can be delivered successfully through a standard cervical incision. However, if this approach is not possible, a median sternotomy is required, and is associated with increased postoperative pain, a prolonged hospital stay and complications in up to 21 per cent of patients^11^, ^12^, ^13^.

Video‐assisted thoracoscopic surgery (VATS) for ectopic mediastinal parathyroid adenoma was first described over 20 years ago^14^, but significant experience has been acquired only in centres performing large numbers of parathyroidectomies. It is an attractive approach for appropriately selected patients, with literature suggesting a shorter hospital stay and lower complication rates^15^, ^16^, ^17^. National Institute for Health and Care Excellence (NICE) guidelines^15^ note that there are limited published data on VATS parathyroidectomy, and recommend the procedure should be performed only in specialist units with a multidisciplinary team.

The majority of patients with biochemically confirmed PHPT who are considering surgery undergo preoperative localization studies^18^. First‐line imaging includes ultrasound imaging of the neck, which has a sensitivity of approximately 60 per cent in parathyroid disease when performed by an experienced operator. However, ultrasonography is unlikely to identify an ectopic mediastinal parathyroid adenoma^19^, ^20^. Technetium‐99 m‐radiolabelled methoxyisobutylisonitrile (MIBI), which can be combined with single‐photon emission CT (SPECT) and low‐dose CT, combines functional and anatomical imaging of parathyroid adenomas, with a sensitivity approaching 97 per cent^21^. An alternative approach is four‐dimensional CT (4DCT): three‐dimensional CT with the added dimension of changes in contrast perfusion over time^22^. 4DCT has been shown to be considerably better than both ultrasound imaging and MIBI/SPECT–CT in localizing parathyroid disease in patients with recurrent or persistent PHPT after previous surgery^23^, ^24^, ^25^, at the expense of a considerably increased dose of radiation^26^.

When ultrasonography, MIBI/SPECT–CT and 4DCT findings are inconclusive, interventional radiology techniques such as selective venous sampling, ideally with angiography, may be considered^27^, ^28^, ^29^, ^30^, ^31^. Recently, [^18^F]fluorocholine PET–CT has been found to identify pathological parathyroid glands^32^, ^33^, ^34^, ^35^, ^36^.

The present study analysed preoperative workup, intraoperative technique and outcomes for this patient series in the context of the existing literature relating to VATS parathyroidectomy.

## Methods

All patients undergoing VATS parathyroidectomy at Hammersmith Hospital from June 2016 to December 2018 were identified from the prospectively developed British Association of Endocrine and Thyroid Surgeons database. Data collected included demographics, preoperative biochemistry and imaging, and the number of previous parathyroid operations. Operative techniques, findings and duration of surgery were documented, as was the postoperative course including biochemistry, length of hospital stay, analgesic requirements, histopathology and complications.

All patients were discussed by a multidisciplinary team involving a radiologist, endocrinologist and endocrine surgeon before listing for surgery. All operations were performed under general anaesthesia, and lung isolation was achieved with a double‐lumen endotracheal tube. Operations were performed by a consultant endocrine surgeon and a consultant cardiothoracic surgeon. A three‐port VATS approach was used, with a 10‐mm optical port and two 5‐mm working ports. If additional retraction was needed, a fourth 5‐mm working port was placed. Insufflation was set at 7 mmHg carbon dioxide. Intraoperative PTH measurement was used in all patients. An underwater seal intercostal drain was placed via the 10‐mm port site at the end of the procedure. All patients had adjusted serum calcium and PTH levels measured on the evening of surgery and again the following morning. The intercostal drain was removed the day after surgery. Patients were discharged once their pain was controlled satisfactorily with oral analgesia. All patients received at least one follow‐up appointment approximately 2 weeks after surgery.

A comprehensive literature review of VATS parathyroidectomy was performed on 1 September 2018 of MEDLINE via PubMed, OvidSP and Science Direct using the search terms ‘parathyroid’, ‘parathyroidectomy’, ‘VATS’, ‘thoracoscopic’, ‘mediastinum’ and ‘mediastinal’. English‐language publications constituted a baseline for inclusion. Non‐clinical studies, published conference abstracts, sporadic case reports and small cohorts with fewer than five patients were excluded. Outcome measures were preoperative localization studies, operative technique, duration of surgery, recovery time, rate of biochemical cure, and the rate and nature of complications, classified as major and minor. Where appropriate, these data were collated.

## Results

Nine patients underwent VATS parathyroidectomy between June 2016 and December 2018 (*Table* 1). Their median age was 55 (range 33–60) years, and six patients were women. All had sporadic PHPT; PHPT was persistent in five patients, and in four the mediastinal parathyroid adenoma was diagnosed before any parathyroid surgery.

**Table 1:** Overview of patient demographics, preoperative blood tests and imaging studies, operative approach and outcomes

	Patient 1	Patient 2	Patient 3	Patient 4	Patient 5	Patient 6	Patient 7	Patient 8	Patient 9
**Diagnosis**	Sporadic PHPT	Sporadic PHPT	Sporadic PHPT	Sporadic PHPT	Sporadic PHPT	Sporadic PHPT	Sporadic PHPT	Sporadic PHPT	Sporadic PHPT
**No. of previous operations**	0	1	3	0	1	0	1	0	2
**PTH (pmol/l)**									
Preoperative	40.9	13.0	15.0	14.1	18.9	9.7	16.2	114.5	25.8
Postoperative*	19.3	2.5	0.5	6.3	3.7	4.8	1.6	0.8	3.0
**Corrected calcium (mmol/l)**									
Preoperative	2.90	2.67	2.76	2.65	2.89	2.80	2.97	3.15	3.12
Postoperative*	2.27	2.11	2.09	2.29	2.29	2.23	2.38	2.62	2.47
**Ultrasound imaging**	Negative	Negative	Negative	Negative	Negative	Negative	Negative	Negative	Negative
**MIBI/SPECT–CT**	Positive	Negative	Negative	Positive	Positive	Negative	Negative	Positive	Negative
**4DCT**	n.d.	Positive	Positive	Not done	Positive	Negative	Positive	Positive	Negative
**Venous sampling**	n.d.	Positive	Positive	Not done	n.d.	Positive	n.d.	n.d.	n.d.
**[** ^**18**^ **F]fluorocholine PET–CT**	n.d.	n.d.	Not done	Not done	n.d.	n.d.	n.d.	n.d.	Positive
**Parathyroid location**	Not found	Intrathymic	Intrathymic	Intrathymic	Intrathymic	Intrathymic	Intrathymic	Left aortopulmonary window	Left aortopulmonary window
**Duration of surgery (min)**	160	60	80	75	99	90	71	120	150
**Histology**	Thymoma	Adenoma	Adenoma	Adenoma	Adenoma	Adenoma	Adenoma	Adenoma	Adenoma
**Weight of parathyroid gland (g)**	–	20.0	13.2	1.6	19.0	7.2	20.6	0.4	0.1
**Complications**	Conversion to sternotomy	None	Transient hypoparathyroidism	None	None	None	None	None	None
**Length of stay (days)**	4	2	1	1	1	2	1	1	2

* Results on day 1 after surgery. PHPT, primary hyperparathyroidism; PTH, parathyroid hormone; MIBI/SPECT, sestamibi/single‐photon emission CT; 4DCT, four‐dimensional CT; n.d., not done.

All patients had ultrasound imaging, which was negative in all, and MIBI/SPECT–CT, which diagnosed two patients correctly. Five patients were diagnosed by 4DCT, two of whom had confirmatory invasive imaging. The limited anatomical information provided by low‐dose SPECT–CT was suggestive, although not conclusive, of a mediastinal abnormality in one patient, who went on to have a negative finding on 4DCT and then positive invasive imaging before successful surgery. Invasive imaging was positive in all three patients in whom it was performed. One patient was diagnosed by [^18^F]fluorocholine PET–CT after a negative 4DCT scan, and did not have invasive imaging.

**Figure 1:**
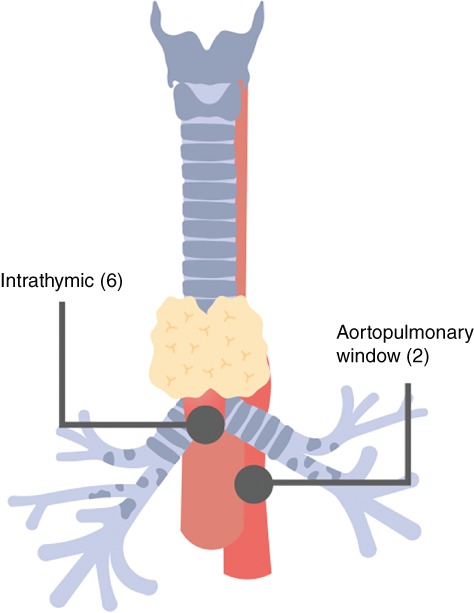
Location of abnormal parathyroid tissue in patients with primary hyperparathyroidism Values in parentheses indicate the number of patients with abnormal tissue at these sites.

The abnormal parathyroid tissue was thought to be intrathymic in seven patients and in the aortopulmonary window in the other two (*Fig*. 1). A left‐sided approach was used in eight patients, and there was a single right‐sided approach.

The median duration of surgery was 90 (range 60–160) min. One patient required conversion to a median sternotomy for removal of a thymoma. All patients returned to the surgical ward after a period of observation in recovery, and no patient required high‐dependency level care. The median length of stay was 1 (range 1–4) days, and seven patients were discharged with simple analgesia only. There were no readmissions within 30 days.

Eight of the nine patients demonstrated biochemical cure. The patient who was not cured was thought to have an intrathymic parathyroid adenoma based on preoperative SPECT–CT. The intraoperative PTH level did not fall after removal of the suspected target lesion. The decision was made to perform a median sternotomy and clear the anterior mediastinum. Intraoperative PTH concentration remained raised, and the decision was made to abandon the procedure. Histopathology confirmed a type AB thymoma, with no parathyroid tissue identified and no PTH markers present. After surgery, the patient became normocalcaemic with a marginally increased PTH level, and remained under regular observation.

In the eight patients who had a biochemical cure, all specimens were reported as hypercellular parathyroid tissue consistent with a parathyroid adenoma. Specimen weights varied considerably, between 0.1 and 20.6 g, depending on the volume of associated thymic tissue removed with the specimen. One patient developed temporary hypoparathyroidism that recovered within 6 months.

The literature review identified nine English‐language case series^16^, ^17^, ^37^, ^38^, ^39^, ^40^, ^41^, ^42^, ^43^ consisting of five or more VATS parathyroidectomies, with a combined total of 87 patients (*Table* 2). PHPT was the most common pathology treated (51 of 69), and 54 per cent of patients (34 of 63) had undergone previous non‐curative neck exploration, consistent with the present patient group. Duration of surgery ranged from 40 to 292 min, and the conversion rate to open surgery was 5 per cent (4 of 87). The most frequent minor complications were transient hypocalcaemia and transient vocal cord palsy. Major complications (Clavien–Dindo grade III–IV^44^) were rare, with an overall rate of 3 per cent (3 of 87). An overall cure rate of 87 per cent (60 of 69) was demonstrated, similar to that in the present series.

**Table 2:** Review of existing literature on video‐assisted thoracoscopic surgical parathyroidectomy

Reference	No. of patients	Sex ratio (M : F)	Age (years)*	Diagnosis	Locali zation method	Previous surgery	Conversion to open operation	Duration of surgery (min)*	LOS (days)*	Rate of cure (%)	Minor complications	Major complications
Alesina *et al*.^37^	7	4 : 3	47 (28–67)	6 PHPT 1 SHPT	CT (100) MIBI (100)	2	0	90 (40–180)	3.8 (2–6)	100	2 (transient hypocalcaemia)	0
Amer *et al*.^38^	7	2 : 5	53 (27–72)	6 PHPT 1 THPT	CT (100) MIBI (57)	3	1	n.s.	2 (1–7)	86	0	0
Du *et al*.^16^	9	n.c.	n.c.	n.s.	CT/MRI (100) MIBI (n.s.)	n.c.	1	68 (46–90)	3.5 (2–5)	n.s.	n.s.	0
Iihara *et al*.^39^	8‡	1 : 7	50 (19–66)	5 PHPT 3 SHPT	CT (100) MIBI (100)	3	0	152 (56–258)	n.c.	75	0	0
Lu *et al*.^40^	12	5 : 7	46 (21–65)	12 SHPT	CT (100) MIBI (100)	12	0	155 (80–292)	5.9 (4–8)	92	6 (3 transient hypocalcaemia, 1 atrial fibrillation, 1 pleural effusion, 1 transient VC palsy)	0
Medrano *et al*.^41^	7	5 : 2	39 (22–57)	6 PHPT 1 SHPT	CT (100) MIBI (100)	7	0	65 (40–92)†	2.7 (2–3)†	100	1 (neuralgia)	0
Randone *et al*.^42^	13	2 : 11	60 (22–88)	13 PHPT	CT (77) MIBI (100) MRI (54) SVS (38)	7	1	92 (50–240)	4.7 (2–15)	77	2 (1 transient VC palsy, 1 pneumonia)	0
Said *et al*.^17^	9	n.c.	n.c.	n.c.	n.c.	0	1	n.c.	n.c.	n.s.	n.c.	2 (1 massive haemothorax, 1 RLN injury)
Wei *et al*.^43^	15	n.c.	n.c.	15 PHPT	CT (100) MIBI (100) SVS (n.c.)	n.c.	0	n.c.	3.3 (n.c.)	87	n.c.	1 (transient VC palsy, 2‐day intubation§)
Overall	87					34 of 63 (54)	4 of 87 (5)			60 of 69 (87)	11 of 54 (20)	3 of 87 (3)

Values in parentheses are percentages unless indicated otherwise;

* values are mean (range) except

† average (range).

‡ Three patients had a combined cervical and thoracoscopic approach; one patient with a sestamibi‐positive thymoma.

§ Pre‐existing right‐sided vocal cord (VC) palsy. LOS, length of stay; PHPT, primary hyperparathyroidism; SHPT, secondary hyperparathyroidism; MIBI, technetium‐99 m‐radiolabelled methoxyisobutylisonitrile sestamibi; n.s., not stated; THPT, tertiary hyperparathyroidism; n.c., not calculable; SVS, selective venous sampling; RLN, recurrent laryngeal nerve.

## Discussion

The key to successful surgical excision of an ectopic mediastinal parathyroid adenoma is accurate preoperative localization. A significant proportion of patients with ectopic mediastinal parathyroid disease will be diagnosed only after a non‐curative bilateral neck exploration.

With persistent PHPT, specialized imaging studies aid in the localization of a target for surgical resection. The target lesion is often small, and may lie within tissue from which it cannot easily be differentiated. This is reflected in the weight of the operative specimens, as intrathymic parathyroid adenomas were resected along with the surrounding thymus, whereas aortopulmonary parathyroid adenomas were removed with minimal surrounding tissue. This is also the second case series to report excision of a thymoma following false‐positive MIBI/SPECT–CT, and surgeons performing mediastinal parathyroidectomy should be aware of this potential pitfall^39^.

As local experience with and confidence in 4DCT increased, this replaced invasive imaging as second‐line investigation of choice. This was reflected in the results, with earlier patients undergoing confirmatory invasive imaging after a positive 4DCT, and later patients undergoing surgery based on positive 4DCT findings alone. Recent access to [^18^F]fluorocholine PET–CT allowed the identification of a target lesion not seen on 4DCT, while avoiding the potential risks to the patient associated with invasive imaging.

Five of the eight patients successfully treated by VATS were discharged home on the first postoperative day, and all VATS patients were discharged home by the second postoperative day. All patients successfully treated by VATS were discharged on only simple analgesia, and none required opioid analgesia after the first few postoperative hours. In comparison, the one patient who required conversion to a median sternotomy was discharged on the fourth postoperative day, and required opioid analgesia at discharge. These results mirror existing reports of comparable patients, undergoing similar preoperative imaging modalities (*Table* 2). Operative duration and conversion rates were similar, although in the present series hospital stay was slightly shorter, with fewer complications than those described in the existing literature.

VATS parathyroidectomy is an effective, safe and well tolerated approach to ectopic mediastinal parathyroid adenoma.

## Disclosure

The authors declare no conflict of interest.
